# Potential of digitalization within physiotherapy: a comparative survey

**DOI:** 10.1186/s12913-022-07931-5

**Published:** 2022-04-13

**Authors:** Katharina Estel, Julian Scherer, Heiko Dahl, Eva Wolber, Noah D. Forsat, David A. Back

**Affiliations:** 1 Clinic for Traumatology and Orthopedics, Bundeswehr Hospital Berlin, Scharnhorststrasse 13, 10115 Berlin, Germany; 2 Department of Traumatology, University Hospital of Zurich, Raemistrasse 100, 8091 Zürich, Switzerland; 3 Physio-Akademie gGmbH Wremen, Wremer Specken 4, 27639 Wuster Nordseeküste, Germany; 4 Medical School of the Charité - Universitätsmedizin Berlin, Charitéplatz 1, 10117 Berlin, Germany

**Keywords:** Physiotherapy, Digitalization, Evaluation, Attitude, Work

## Abstract

**Background:**

Due to the global digitalization, implementation of digital elements into daily work can support physiotherapists’ work but may also pose some challenges. Only little is known about physiotherapists’ attitude towards digitalization. This study primarily aimed to analyze physiotherapists’ attitude towards digitalization and to what extend digital tools have been implemented into their daily work. In second analysis, participants’ characteristics such as age, working place, gender and mode of survey participation were assessed.

**Methods:**

A 12-main-item survey amongst voluntary course participants of one physiotherapeutic training center was conducted via paper-based as well as online questionnaires between July 2018 and June 2019 including questions on participants’ general as well as particular attitude towards digitalization, the use of (mobile) applications and possible advantages and disadvantages of the ongoing digital transformation. Sub-analysis was performed for age (≤40 years versus > 40 years), gender, mode of participation (paper vs. online) and working place (practice vs. hospital).

**Results:**

Overall, 488 physiotherapists participated in the survey. In comparison of the age groups, younger participants had more concerns about data security (*p* = 0.042) and insufficient financial remuneration (*p* < 0.001). Younger participants stated higher satisfaction with data literacy than their counterparts (*p* = 0.0001). Physiotherapists working in the outpatient sector, rather than in hospitals, expected digitalization to increase more in relevance (*p* < 0.001). The online respondents (OG) indicated that they had more knowledge about key aspects of the current legal situation regarding digitalization than participants completing the paper-based survey (*p* = 0.002). 50.4% of the considered digitalization as useful for their job.

**Conclusions:**

The majority of participants saw high potential for digitalization in the physiotherapy sector. Younger physiotherapists seem to be more concerned about data security and insufficient financial remuneration. Physiotherapists in the outpatient sector seem to see more potential in digital transformations. General concerns like missing reimbursement, lack of data security or knowledge on legal frameworks should be addressed in the future. Further studies should focus on identifying specific digital tools which can support physiotherapists.

**Supplementary Information:**

The online version contains supplementary material available at 10.1186/s12913-022-07931-5.

## Background

The current digital transformation can be seen as a central topic of our modern society and is also affecting the healthcare sector to a vast extend [1]. While some medical disciplines, such as radiology, have already been highly influenced by digital innovations, others with need for a physical examination, such as orthopedics, are rather in an early stage of digital adaptation [2]. As a healthcare discipline with close and intense patient contact, but also with many aspects of transportable digital knowledge, physiotherapy is also increasingly affected by digital influences [3, 4].

The use of electronic documentation can increase work efficiency by standardizing work processes and can help monitoring measured physical parameters such as the range of motion (ROM) [5]. In the context of telehealth, studies have shown, that a physiotherapeutic assessment of the knee with digital tools (e.g., telehealth) appears to be feasible and reliable [6, 7]. Furthermore, telehealth offers the possibility to reach many patients and to reduce costs while being also available for patients in remote areas [8–11]. For surgical patients, telehealth has been shown to be as effective as usual hands-on care in specific settings [12, 13]. Therefore, telehealth can have a positive impact on health outcomes and patients’ satisfaction [5, 9, 10]. As another relevant digital field, mobile health applications (mHealth) have been reported to support physiotherapeutical treatment with a high level of patient satisfaction [14]. In combination with mobile applications, wearable devices can be successfully used for real-time and comprehensive patient monitoring [15]. Positive attitudes towards mHealth instruments amongst physiotherapists have been shown in a previous study [16]. On the other hand, several studies have shown that patients as well as physiotherapists are generally concerned about data security, which seems to remain a disadvantage of digitalization [17–19].

Besides many promising digital devices and procedures, the field of physiotherapy has some challenges to face [5]. For example, certain groups of patients, such as children or elderly people, may have trouble using telehealth [20]. Furthermore, in some countries, digital practices are not recognized by health-insurance companies, and therefore are not adequately reimbursed [5, 21, 22]. However, during the rapid spread of digitalization within the healthcare system, the legal framework can be expected to be further adapted.

Especially during the SARS-CoV-19 pandemic, digitalization has gained enormous importance [23]. Several studies have shown an increased use of telehealth due to governmental measurements and in order to protect staff as well as patients, underlining the need for new digital tools [24, 25].

The present study aimed to identify physiotherapists’ attitude towards digitalization and how far digital features have already found their way into their everyday work before the Covid-19 pandemic. Furthermore, we aimed to identify factors contributing to participants’ attitudes such as gender, working place, age and – as potential bias [26, 27] – online versus hard-copy participation (mode of participation).

## Methods

### Study design

A survey amongst physiotherapists was conducted at a large physiotherapeutic training center with participants from all over Germany (Physio-Akademie gGmbH Wremen, Wurster Nordseeküste, Germany). The Physio-Akademie is an educational institution and scientific institute offering continuous education and training, research as well as development in physiotherapy. It cooperates with universities and scientific institutions and offers classes and online courses for physiotherapists. In 2021, approximately 3.200 participants from all over Germany attended courses, making the Physio-Akademie one of Germany’s biggest physiotherapeutic training institutions. The survey was conducted from July 2018 to June 2019 among course participants. The participants were asked to either complete the survey online using SurveyMonkey® (SurveyMonkey Inc., Oregon, USA) (online group = OG) or using hard copies (presence group = PG). The allocation of the participants to one of the two modes of survey completion was random. Further subgroups were formed for age (≤40 years versus > 40 years, arbitrary threshold), working place (outpatient sector versus hospital sector) and gender (female versus male).

Participation was voluntary and anonymity was granted. All participants received a written information explaining the aim of the study and processing of their data. By answering the questionnaire, participants gave consent to the use of the data that they had provided. Ehtical approval was granted by the local ethics committee.

### Questionnaire

The questionnaire was developed based on a non-published questionnaire for orthopedic and trauma doctors. The further literature backgrounds were a German survey conducted by the German medical association “Marburger Bund” among 1800 employed physicians in september/october 2017 on the topic of digitalization [28] and a survey by Blumenthal et al. among 76 physiotherapists in Canada [16]. The questionnaire was validated by a group of physiotherapists and physicians (KE, JS, HD, EW, NDF, DAB). The final questionnaire consisted of 12 questions, aiming to assess the attitude and the use of digital tools amongst physiotherapists. The questions were divided into three groups that addressed the following areas of interest:

1. Sociodemographic data (age, gender) and place of employment (three questions).
2. Attitude towards digitalization and the use of digital features: Participants’ opinion, own digital knowledge (= data literacy), use of applications, the perceived potentials and pitfalls of digitalization, the importance of “Big Data” or “artificial intelligence”, the use of digital services in the work environment and the existing knowledge about essential aspects of the current legal situation regarding digitalization (eight questions).
3. In a concluding open question, participants were able to report comments (one question).

In three questions, where rating scales were used, the number “1” was set as the highest positive consent.

### Data analysis

For statistical analysis, SPSS (version 27.0, IBM Corp., Armonk, NY, USA) was used. Non-parametric median-test was applied for analysis of non-categorical data. Categorical data were analyzed by chi-square test. A subgroup analysis was performed for the above-mentioned topics, age, gender, working sector and online versus hard copy participation. The level of significance was set at *p* < 0.05. The free-text answers were analyzed by two independent experts for repetitive sequences. A systematic rule-driven qualitative text analysis was performed using techniques of qualitative content analysis according to Mayring: the free-text responses were selected from the questionnaires and examined for essential question content; a summary was performed to reduce the responses to a short text, and the summaries were analyzed; the results were interpreted; and a quality analysis was performed to ensure that the appropriate criteria were met [29].

## Results

### Sociodemographic data

A total of 488 physiotherapists participated in the survey (167 male, 314 female, 7 missing, 1.4%). Of all participants, 263 (53.9%) conducted the survey online and 225 (46.1%) on paper sheets. 269 (55.1%) of the participants were 40 years of age or younger, compared to 197 (40.4%) participants with an age of more than 40 years (22 missing, 4.5%). 54 (11.1%) participants were from the hospital sector, whereas 427 (87.5%) were employed in the outpatient sector (7 missing, 1.4%).

### Opinion on digitalization

50.4% (*n* = 246) of all participants stated (rating scale 1–5), that digitalization was interesting and that they would use it, if any benefit was seen. No statistical differences were assessed for gender, mode of participation, participants’ age groups or job assignments. Further results are shown in Table 1.

**Table 1:** Results of the surveyed participants (*n* = 488) for the comparisons of male vs. female, age (22 missing), hospital sector (= HS) vs. outpatient sector (= OS) (7 missing) and online group (= OG:) vs. presence group (= PG). Individual *p*-values are shown for each comparison. Individual number of total answers are given for each question (with possible abstention option in the questionnaire) as well as total participation numbers

Questions and possible answers	Total group n	statistically significant differences (***p*** < 0.05) between …											
		Younger than 40 years (Y) vs. Older than 40 years (O)			male (m) vs. female (f)			Hospital Sector (HS) vs. Outpatient Sector (OS)			Online Group (OG) vs. Presence Group (PG)		
		***Y***	***O***	***p***	***m***	***f***	***p***	***HS***	***OS***	***p***	***OG***	***PG***	***p***
***What do you think about digitalization?***	***454***	***n = 256 (%)***	***n = 184 (%)***	*0.100*	***n = 153 (%)***	***n = 301 (%)***	*0.860*	***n = 51 (%)***	***n = 403 (%)***	*0.065*	***n = 239 (%)***	***n = 217 (%)***	***0.178***
I find the topic very interesting and participate actively involved		35 (14)	30 (16)		32 (21)	38 (13)		12 (23)	57 (14)		54 (23)	16 (8)	
The topic is interesting. I use offers if they fit into my profession.		152 (59)	91 (49)		73 (48)	171 (56)		29 (57)	216 (54		105 (44)	141 (65)	
I wait with a use, until more experiences are present.		47 (18)	34 (19)		33 (21)	52 (17)		6 (12)	79 (20)		41 (17)	44 (20)	
I am rather skeptical about this topic.		18 (7)	18 (10)		10 (7)	29 (10)		2 (4)	37 (9)		26 (11)	13 (6)	
I would rather not include this topic in my job integrate.		4 (2)	11 (6)		5 (3)	11 (4)		2 (4)	14 (3)		13 (5)	3 (1)	
***How “fit” do you keep yourself when it comes to digitalization in relation to your professional activities? (Indication by school grades)***	***461***	***n = 258***	***n = 187***	***0.0001***	***n = 154***	***n = 305***	*0.379*	***n = 51***	***n = 408***	*0.057*	***n = 240***	***n = 221***	*0.142*
Very good (“1”)		19 (7)	9 (5)		19 (12)	12 (3)		5 (10)	25 (6)		21 (9)	10	
Good (“2”)		80 (31)	46 (24)		59 (38)	72 (24)		17 (33)	113 (28)		68 (28)	63	
Satisfying (“3”)		111 (43)	62 (33)		39 (25)	136 (45)		21 (41)	155 (38)		80 (33)	96	
Sufficient (“4”)		38 (15)	50 (27)		29 (20)	62 (20)		6 (12)	86 (21)		52 (22)	40	
Defective (“5”)		8 (3)	17 (9)		5 (3)	21 (7)		1 (2)	25 (6)		16 (7)	10	
Insufficient (“6”)		2 (1)	3 (2)		3 (2)	2 (1)		1 (2)	4 (1)		3 (1)	2	
***Do you use smartphone apps in your professional life? (multiple answers possible)***	***488***	***n = 269***	***n = 197***		***n = 167***	***n = 314***		***n = 54***	***n = 427***		***n = 263***	***n = 225***	
Yes - for my own organizational support (e.g. patient calendar or similar).		52 (19)	46 (23)	0.293	42 (25)	57 (18)	0.071	9 (17)	90 (21)	0.450	58 (22)	41 (18)	0.294
Yes - to my professional support (e.g. AO app, apps for specific training methods, or similar).		76 (28)	55 (28)	0.937	48 (29)	87 (28)	0.810	14 (26)	120 (28)	0.737	74 (28)	61 (27)	0.801
Yes - for communication with colleagues.		161 (60)	85 (43)	**0.000**	83 (50)	168 (54)	0.427	29 54)	221 (52)	0.787	113 (43)	139 (62)	**0.000**
Yes - for communication with patients (e.g. own practice app, apps for therapy documentation, etc.)		29 (11)	28 (14)	0.264	19 (11)	42 (13)	0.531	1 (2)	60 (14)	**0.011**	19 (7)	42 (19)	**0.012**
No, I don’t want any apps for use my physiotherapeutic activity.		39 (14)	54 (27)	**0.001**	34 (20)	61 (19)	0.807	13 (24)	83 (19)	0.422	60 (23)	36 (16)	0.059
***What potential do you associate with “digitalization” in physiotherapy? (multiple answers possible)***	***488***	***n = 269***	***n = 197***		***n = 167***	***n = 314***		***n = 54***	***n = 427***		***n = 263***	***n = 225***	
Patient care will be improved.		74 (28)	51 (26)	0.696	52 (31)	76 2 (4)	0.101	15 (28)	112 (26)	0.808	77 (29)	52 (23)	0.124
My work is made easier (e.g. organization, procedures and processes...).		197 (73)	121 (61)	**0.007**	105 (63)	223 (71)	0.068	39 (72)	289 (68)	0.500	167 (63)	163 (72)	**0.035**
Communication among colleagues is made easier.		177 (66)	99 (50)	**0.001**	95 (57)	188 (60)	0.526	31 (57)	252 (59)	0.821	138 (52)	146 (65)	**0.006**
Communication with patients is made easier.		91 (34)	62 (31)	0.593	67 (40)	93 (30)	**0.020**	15 (28)	144 (34)	0.382	92 (35)	68 (30)	0.264
Disease prevention measures are being expanded.		67 (25)	48 (24)	0.893	39 (23)	78 (25)	0.717	14 (26)	102 (24)	0.741	68 (26)	49 (22)	0.293
Rehabilitation measures are being expanded.		63 (23)	48 (24)	0.813	38 (23)	74 (24)	0.841	16 (30)	95 (22)	0.225	72 (27)	41 (18)	**0.017**
***What potential problems do you associate with “digitalization” in physiotherapy? (multiple answers possible)***	***488***	***n = 269***	***n = 197***		***n = 167***	***n = 314***		***n = 54***	***n = 427***		***n = 263***	***n = 225***	
The effort of introducing digital technologies is high		109 (41)	103 (52)	**0.012**	86 (51)	135 (43)	0.075	27 (50)	195 (46)	0.547	128 (49)	94 (42)	0.128
The physiotherapist-patient relationship is worsened.		26 (10)	20 (10)	0.862	14 (8)	33 (11)	0.455	7 (13)	40 (9)	0.402	26 (10)	21 (9)	0.837
The practical implementation is not yet mature enough.		83 (31)	62 (31)	0.887	52 (31)	99 (32)	0.930	12 (22)	139 (33)	0.123	77 (29)	74 (33)	0.390
I myself have too little experience with the topic.		74 (28)	75 (38)	**0.016**	27 (16)	123 (39)	**0.000**	16 (30)	136 (32)	0.741	81 (31)	71 (32)	0.857
Data protection is a relevant issue.		137 (51)	119 (60)	**0.042**	92 (55)	172 (55)	0.984	26 (48)	237 (56)	0.306	155 (59)	109 (48)	**0.020**
Financial compensation as an incentive is still insufficient		84 (32)	103 (52)	**0.000**	69 (41)	124 (39)	0.697	20 (37)	173 (41)	0.623	142 (54)	51 (23)	**0.000**
***How do you estimate the importance of “Big Data” or “artificial intelligence” in your profession***	***438***	***n = 245***	***n = 178***	*0.653*	***n = 149***	***n = 287***	*0.777*	***n = 51***	***n = 385***	***0.001***	***n = 233***	***n = 205***	*0.431*
Both will significantly shape the way I work.		18 (7)	6 (3)		15 (10)	9 (3)		7 (14)	16 (4)		15 (6)	9 (4)	
The topic will increase in relevance.		140 (57)	105 (60)		79 (53)	176 (61)		36 (70)	220 (57)		130 (56)	126 (62)	0.525
The topic will not play a relevant role in physiotherapy.		41 (17)	27 (15)		31 (21)	39 (14)		4 (8)	65 (17)		37 (16)	33 (16)	
The topic will be irrelevant for my personal work.		46 (19)	40 (22)		24 (16)	63 (22)		4 (8)	84 (22)		51 (22)	37 (18)	
***Which of the following digital services are already in use today by you or your employer for your work area? (multiple answers possible)***	***488***	***n = 269***	***n = 197***		***n = 167***	***n = 314***		***n = 54***	***n = 427***		***n = 263***	***n = 225***	
Electronic patient file		95 (35)	69 (35)	0.948	60 (36)	106 (34)	0.634	37 (69)	128 (30)	**0.000**	93 (35)	74 (33)	0.566
Online video consultation		1 (1)	5 (3)	**0.040**	5 (3)	1 (1)	**0.012**	1 (2)	5 (1)	0.671	6 (2)	0 (0)	**0.023**
Your own homepage		215 (80)	138 (70)	**0.014**	127 (76)	236 (75)	0.829	34 (63)	329 (77)	**0.023**	183 (70)	182 (81)	**0.004**
Electronic appointment allocation		72 (27)	43 (22)	0.222	47 (28)	71 (23)	0.179	17 (31)	100 (23)	0.193	52 (20)	66 (29)	**0.014**
Telemedicine (e.g. video-based communication with colleagues/doctors/patients or similar)		7 (3)	6 (3)	0.774	7 (4)	7 (2)	0.223	4 (7)	10 (2)	**0.037**	11 (4)	3 (1)	0.060
Social media (e.g. Facebook/Instagram/Twitter or similar)		106 (39)	51 (26)	**0.002**	66 (40)	96 (31)	**0.048**	11 (20)	150 (35)	**0.030**	78 (30)	84 (37)	0.073
Email contact possibility		205 (76)	141 (72)	0.258	129 (77)	230 (73)	0.337	36 (67)	323 (76)	0.153	192 (73)	169 (75)	0.597
***Would you claim that you are aware of essential aspects of the current legal situation regarding digalitization (including national e-health laws, European Data Protection Regulation,*** **etc.*)?***	***443***	***n = 249***	***n = 178***	*0.085*	***n = 146***	***n = 296***	*0.353*	***n = 47***	***n = 394***	*0.685*	***n = 239***	***n = 204***	***0.002***
I fully agree		5 (2)	10 (6)		3 (2)	13 (4)		1 (2)	15 (4)		12 (5)	4 (2)	
Rather yes		60 (24)	63 (35)		58 (40)	71 (24)		13 (28)	115 (29)		91 (38)	38 (19)	
Rather no		153 (62)	92 (52)		67 (46)	184 (62)		29 (62)	223 (57)		121 (50)	131 (64)	
Not at all		31 (12)	13 (7)		18 (12)	28 (10)		4 (8))	41 (10)		15 (7)	31 (15)	

### Data literacy

On a given scale from 1 (very good) to 6 (insufficient), one third (*n* = 176 (36.1%), 27 missing) stated that they were satisfied with their data literacy (mean 2.92, SD 1.03). Younger participants rated a higher satisfaction than older ones (*p* = 0.0001). Further results are shown in Table 1.

### Smartphone app usage

In regards of mobile app usage, 99 (20.3%) participants stated that they used it for their own organizational support, 135 (27.7%) for their professional support, 252 (51.6%) for communication with colleagues, 61 (12.5%) for communication with patients (e.g., own practice app, apps for therapy documentation, etc.) and 96 (19.7%) participants indicated that they did not want to use any apps for work purposes. Significant differences for the comparisons of age groups, mode of participation, and job assignments are shown in Table 1.

### Potential of digitalization

**Fig. 1:**
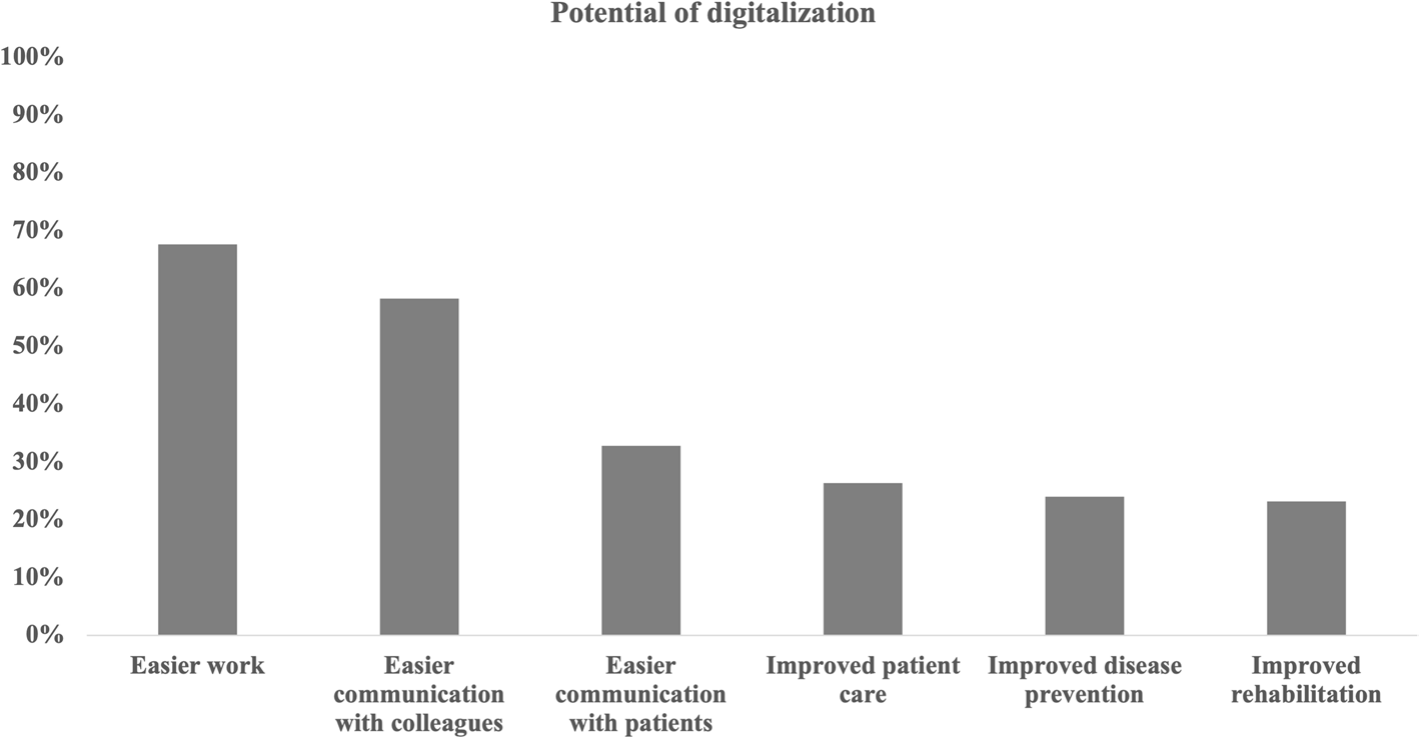
Potential of digitalization within the field of physiotherapy estimated by the survey participants (*n* = 488; multiple answers possible)

**Fig. 2:**
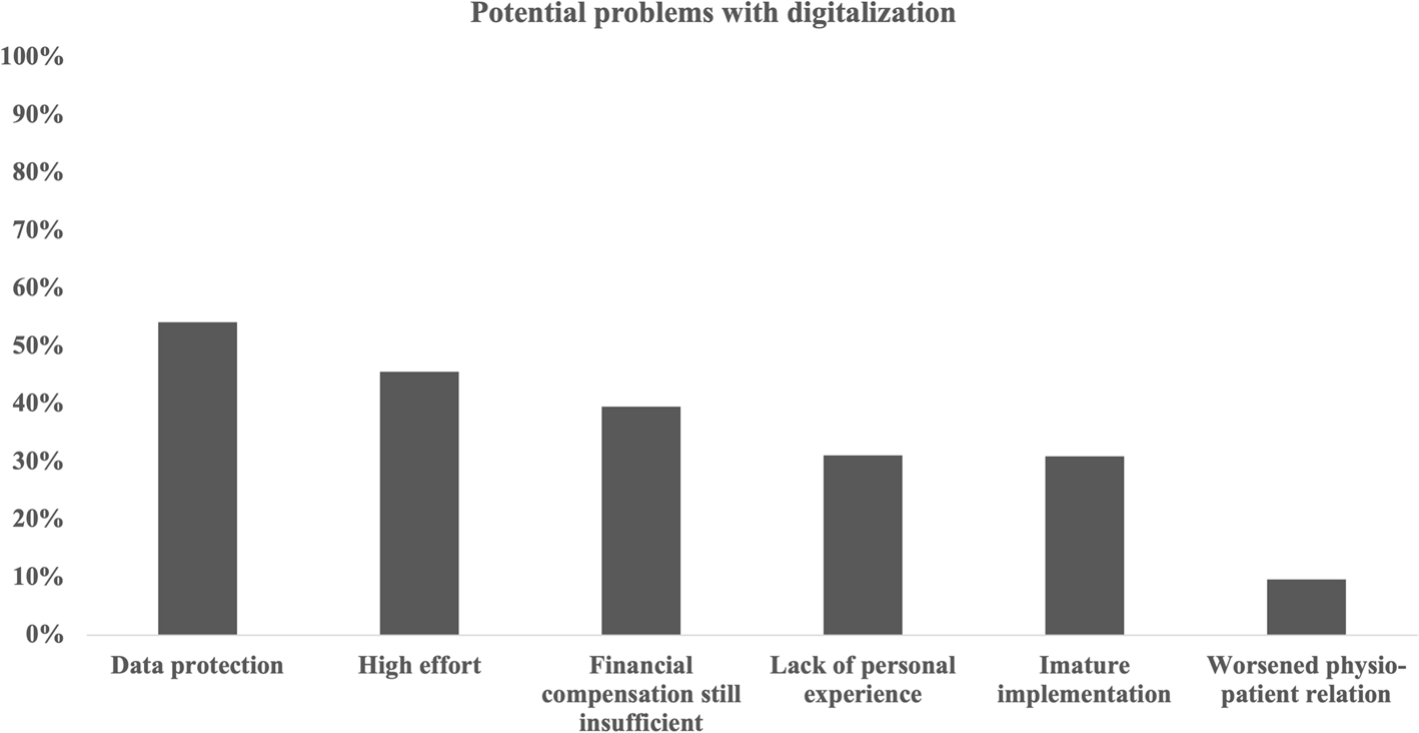
Potential problems associated with digitalization within the field of physiotherapy by the survey participants (*n* = 488; multiple answers possible)

With regards to the potential of digitalization (with multiple answers possible) most participants stated that digitalization could make work easier (67.6%), followed by better communication between colleagues (58.2%) and with patients (32.8%) [Fig. 1]. Differences between the sub-groups are shown in Table 1. Figure 2 shows an overview of the potential problems reported by the participants, with most answers agreeing on data protection concerns (*n* = 264, 54.1%), followed by concerns about the high effort regarding a successful implementation of new digital tools (*n* = 222, 45.5%) and concerns about insufficient financial compensation (*n* = 193, 39.5%). Further results are shown in Table 1.

### Importance of big data or artificial intelligence

When asked about the importance of Big Data or artificial intelligence (AI) (rating scale 1–4), 24 (4.9%), physiotherapists indicated that both will significantly shape the way of their work and 256 (52.5%) stated that this topic will increase in relevance. 70 (14.3%) of the participants stated that Big Data or AI will not play a significant role in physiotherapy and 88 (18.0%) stated that both will be irrelevant for their work (50 answers missing, 10.2%). Further results are shown in Table 1.

### Digital services in use

**Fig. 3:**
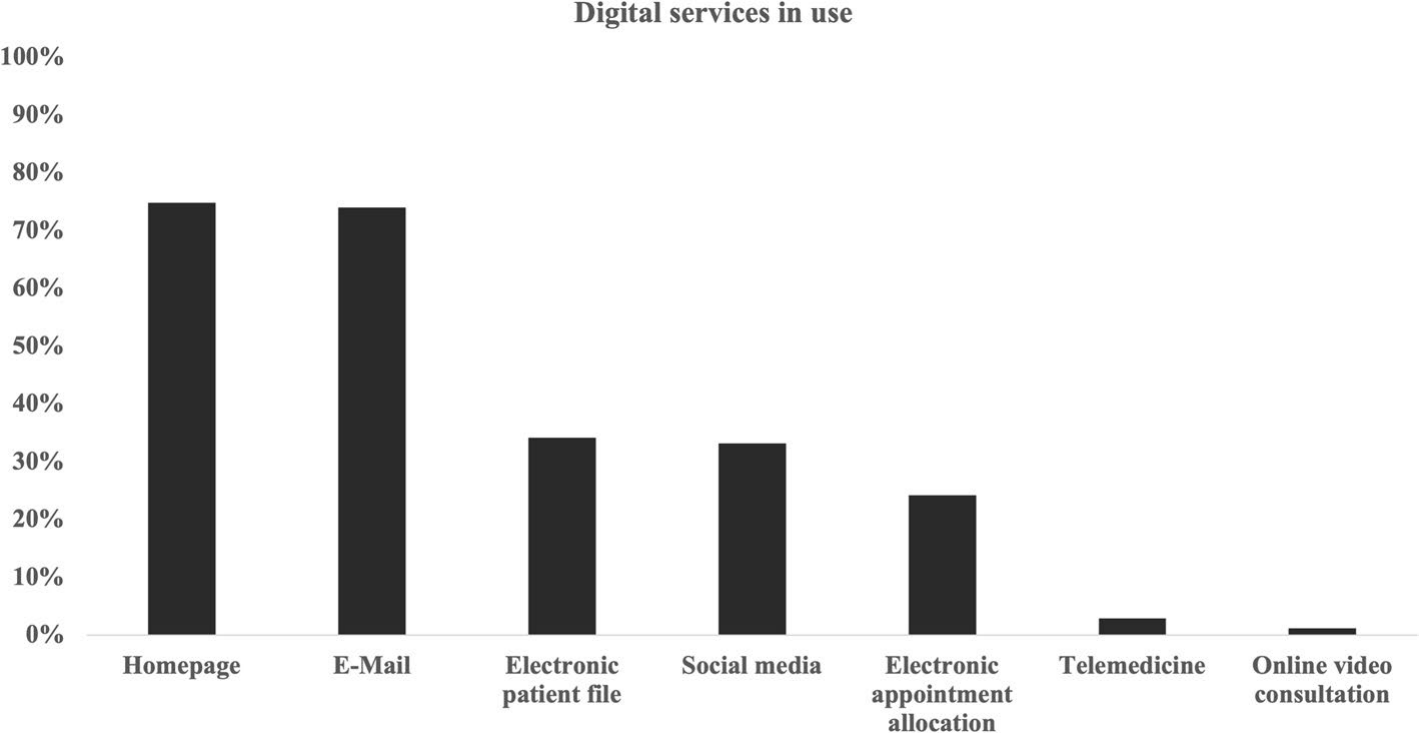
Digital services already used by the survey participants (*n* = 488; multiple answers possible)

When asked about digital services already used for their work, 74.8% (*n* = 365) indicated that they were using a website and 74% (*n* = 361) were using emails. Only 6 participants (1.2%) were using online video consultations [Fig. 3]. Further results are shown in Table 1.

### Awareness of current legal situation

The majority of the surveyed physiotherapists (*n* = 252, 45 missing) stated that they were rather unaware of key aspects of the current legal situation regarding digitalization (mean 2.74, SD 0.68) (rating scale 1–4, with one being aware and 4 being completely unaware). PG participants were lesser informed than OG (*p* = 0.002). Further results are shown in Table 1.

### Free comments on “digitalization”

Most comments focused on the wish to uniform laws and an implementation of the acquisition of data literacy in training/studies. In addition, the hope for easier cooperation with other professional groups in the future was mentioned. Concerns were lacking data security and financial remuneration, and that contact with patients could change negatively.

## Discussion

With the current digital transformation of the healthcare system, also physiotherapy is increasingly affected by new developments [3, 4, 30]. Various elements like electronic documentation, telehealth and mHealth can make physiotherapists’ daily work easier and increase their efficiency [5, 6, 14]. Thus, the primary aim of the present study was to investigate the attitude towards different aspects of digitalization amongst physiotherapists. Furthermore, a sub-analysis of participants’ age, workplace, gender, and mode of survey participation aimed to assess factors contributing to the surveyed physiotherapists’ attitudes.

Half of all participants stated that digitalization was interesting and that they would use elements of it, if it suited their work. In addition, most of the participants considered the potential of digitalization very high. This result is consistent with findings of a previous study, which showed that the work of physiotherapists can be made easier and more efficient by implementing digital tools [5]. Younger participants agreed with this statement significantly more often than their elderly counterparts. Furthermore, this group also stated significantly more often that communication with colleagues would be more feasible compared to the older group of participants. To our knowledge, this facet of digitalization has not yet been investigated in other studies, but a stronger affinity to digitalization among the younger generation of physiotherapists [31].

Websites and e-mails were the most frequently utilized digital services in the surveyed participants. Only a few physiotherapists, especially the participants over 40 and those working in the outpatient sector, had used online video consultations. This is unexpected since several studies have shown that telehealth can be equivalent to conventional treatments, and other health disciplines are already using this digital tool [12, 13, 24, 25]. This finding suggests that several digital tools have not yet been implemented widely amongst physiotherapists [32] and might go along with the findings presented in this study, that only one third of those surveyed stated, that they were satisfied with their data literacy. To our knowledge, there is no comparative data in the literature on digital skills for physiotherapists and possible reasons for this finding remain unclear. However, a survey of nurse trainees on eHealth skills showed that 45% of respondents were satisfied with their internet skills [33]. E-Health can be referred to as the use of information and communications technologies in support of health and health-related fields [34]. Nevertheless, another study on eHealth competence among college students revealed a relatively high percentage of incorrect self-assessment for these abilities [35], so that the presented results should be examined further in future studies.

Almost one fifth of physiotherapists in the present study had not yet used digital applications in their everyday work. The literature also showed that mHealth is not yet considered an integral part amongst health care professionals and especially not physiotherapists [36, 37], although several studies have proved advantages of digital tools regarding patients’ satisfaction [14, 38]. Insufficient knowledge and low experience were identified as potential causes of low digital usage in health professions in a previous study [37]. Other authors reported that available mHealth tools did not meet physiotherapists’ usage expectations [16].

Most of the participants in the present study stated, that they used digital tools to organize their own work or practice. This can be seen as promising for the future, since the use of mHealth in surgical patients, in the peri- and postoperative phases, showed to have a positive effect and treatment plan adherence [39].

Amongst several positive aspects of digitalization, this study also assessed concerns of the surveyed participants. Previous studies have identified lacking security and privacy of data as potential disadvantages [40, 41], which were also the main concern in the present study. Younger participants as well as participants in the online survey stated these concerns more frequently than their counterparts. Furthermore, especially younger physiotherapists had concerns about the integration of new technologies into existing systems, which is consistent with findings of a previous study [40].

Like in all other areas, digitalization within physiotherapy also includes the topics “Big Data” and “artificial intelligence”, which are becoming increasingly important [42]. Large-scale patient-related data analysis can successfully help developing new treatment strategies [32, 43]. Furthermore, with the help of personal data, clinical records, exercise evaluations and videos, physiotherapists may receive support for the assessment and evaluation of treatment results [40]. Most of the physiotherapists in the present cohort also considered these topics important for their future work. However, nearly 20% of respondents said that Big Data and AI were not increasingly relevant to them. Female physiotherapists and physiotherapists working in the outpatient sector considered this topic to be less relevant. To the author’s believe, possible reasons for this phenomenon are lack of data, lack of experience in using digital tools [37], or reluctance to include digital tools into one’s work.

In our study, most physiotherapists stated that they were unaware of key aspects of the current legal situation regarding digitalization. In Germany, the first newer legislative initiatives on digitalization of the healthcare system by the government have taken place since 2015 and have been significantly expanded since then [43]. Thus, the attitude and engagement of physiotherapists to digitalization might have changed till today, especially also in the context of the current Covid-19 pandemic. Yet, it also must be taken into consideration that political goals and strategies for implementing digitalization may vary between countries and hence also physiotherapists’ attitudes in a multinational comparison*.* Amongst the few who stated to have better knowledge of this, physiotherapists over 40 years, and online respondents were predominant. For the group of elderly participants, this could be due to greater work experience. There are no existing comparative studies for physiotherapists in this regard. However, the legal situation regarding digitalization within the healthcare system may vary between national and international levels [44, 45]. This could make it more difficult for an individual user to build up knowledge about the key aspects of the current legal situation. Physiotherapeutic associations and societies can play a front role for providing information and orientation to address this issue.

The study is limited by the number of participants compared to the number of active physiotherapists in Germany, making it not representative. Therefore, − especially in the current situation with the Sars-CoV-19 pandemic – the presented data should be further validated in multi-center studies with a larger sample size and also in direct relation to measurable improvements of digital tools in medicine. In this context, another limitation is the self-reporting character and thus subjective source of the current data, making a more objective data generation necessary. Since the presented results must be considered incongruent, this study cannot give deduction on a relevant bias of online versus paper-based surveys on digital topics. Another serious limitation of this study was that a definite survey response rate could not be stated, since the exact number of recipients was distorted due to unclear numbers of not-received emails and absences in the courses at the day of evaluation.

A further limitation is the fact that the survey participation in a paper-based or online mode was defined by the authors, which may have influenced the outcome. Additionally, questions on the potential advantages as well as potential problems of digitalization, had predefined answer options. This means that the entire range of answers was most likely not fully covered, which should be addressed in further surveys by adding a free-text comment option. Furthermore, we conducted a post-hoc-power analysis for the working-place subgroup, which revealed inhomogeneous results ranging from 9.8 to 87.1%.

## Conclusion

The majority of participants saw high potential for digitalization in the physiotherapy sector. Younger physiotherapists seem to be more concerned about data security and insufficient financial remuneration but showed higher digital affinity and a significant higher satisfaction with their data literacy. Physiotherapists in the outpatient sector seem to see more potential in digital transformations. General concerns like missing reimbursement, lack of data security or knowledge on legal frameworks should be addressed in the future. Further studies should focus on identifying specific digital tools which can support physiotherapists, preferably in close cooperation with active physiotherapists to enhance acceptance within the physiotherapeutical society.

## Supplementary Information

**Additional file 1.**

## Data Availability

The datasets generated during and analyzed during the current study are not publicly available since the authors did not obtain consent from the participants to publish raw data publicly but are available from the corresponding author on reasonable request.

## References

[1] Wolf B, Scholze C. Medicine 4.0. Curr Dir Biomed Eng. 2017;3:183–6.

[2] Harren K, Dittrich F, Reinecke F, Jäger M (2018). Digitalization and artificial intelligence in orthopedics and traumatology. Orthopade.

[3] Calvaresi D, Calbimonte J-P (2020). Real-time compliant stream processing agents for physical rehabilitation. Sensors (Basel).

[4] Peolsson A, Landén Ludvigsson M, Peterson G (2017). Neck-specific exercises with internet-based support compared to neck-specific exercises at a physiotherapy clinic for chronic whiplash-associated disorders: study protocol of a randomized controlled multicentre trial. BMC Musculoskelet Disord.

[5] Digital Physical Therapy Task Force, Lee A, Finnin K, Holdsworth L, Millette D, Peterson C. Report of the wcpt/inptra digital physical therapy practice task force. 2019. https://world.physio/sites/default/files/2021-06/digital-practice-report-2021-FINAL.pdf.

[6] Richardson BR, Truter P, Blumke R, Russell TG (2016). Physiotherapy assessment and diagnosis of musculoskeletal disorders of the knee via telerehabilitation. J Telemed Telecare.

[7] Lamplot JD, Pinnamaneni S, Swensen-Buza S, Lawton CD, Dines JS, Nawabi DH (2020). The virtual shoulder and knee physical examination. Orthop J Sports Med.

[8] Bossen D, Veenhof C, Van Beek KE, Spreeuwenberg PM, Dekker J, De Bakker DH (2013). Effectiveness of a web-based physical activity intervention in patients with knee and/or hip osteoarthritis: randomized controlled trial. J Med Internet Res.

[9] Grona SL, Bath B, Busch A, Rotter T, Trask C, Harrison E (2018). Use of videoconferencing for physical therapy in people with musculoskeletal conditions: a systematic review. J Telemed Telecare.

[10] Levy CE, Silverman E, Jia H, Geiss M, Omura D (2015). Effects of physical therapy delivery via home video telerehabilitation on functional and health-related quality of life outcomes. J Rehab Res Dev.

[11] Kloek CJJ, van Dongen JM, de Bakker DH, Bossen D, Dekker J, Veenhof C (2018). Cost-effectiveness of a blended physiotherapy intervention compared to usual physiotherapy in patients with hip and/or knee osteoarthritis: a cluster randomized controlled trial. BMC Public Health.

[12] van Egmond MA, van der Schaaf M, Vredeveld T, Vollenbroek-Hutten MMR, van Berge Henegouwen MI, Klinkenbijl JHG (2018). Effectiveness of physiotherapy with telerehabilitation in surgical patients: a systematic review and meta-analysis. Physiotherapy.

[13] Pastora-Bernal JM, Martín-Valero R, Barón-López FJ, Moyano NG, Estebanez-Pérez M-J (2017). Telerehabilitation after arthroscopic subacromial decompression is effective and not inferior to standard practice: preliminary results. J Telemed Telecare.

[14] Stütz T, Emsenhuber G, Huber D, Domhardt M, Tiefengrabner M, Oostingh GJ (2017). Mobile phone–supported physiotherapy for frozen shoulder: feasibility assessment based on a usability study. JMIR Rehabil Assist Technol.

[15] Vallati C, Virdis A, Gesi M, Carbonaro N, Tognetti A (2018). ePhysio: a wearables-enabled platform for the remote management of musculoskeletal diseases. Sensors (Basel).

[16] Blumenthal J, Wilkinson A, Chignell M (2018). Physiotherapists’ and physiotherapy students’ perspectives on the use of mobile or wearable technology in their practice. Physiother Can.

[17] Kuhn S, Kadioglu D, Deutsch K, Michl S. Data literacy in der medizin: welche kompetenzen braucht ein arzt? Onkologe. 2018;24.

[18] Hege I, Tolks D, Kuhn S, Shiozawa T (2020). Digital skills in healthcare. GMS J Medical Edu.

[19] Scherer J, Keller F, Pape H-C, Osterhoff G (2020). Would patients undergo postoperative follow-up by using a smartphone application?. BMC Surg.

[20] Kruse C, Fohn J, Wilson N, Nunez Patlan E, Zipp S, Mileski M (2020). Utilization barriers and medical outcomes commensurate with the use of telehealth among older adults: systematic review. JMIR Med Inform.

[21] Lee AC, Davenport TE, Randall K (2018). Telehealth physical therapy in musculoskeletal practice. J Orthop Sports Phys Ther.

[22] Bierman RT, Kwong MW, Calouro C (2018). State occupational and physical therapy telehealth Laws and Regulations: a 50-state survey. Int J Telerehabil.

[23] Keesara S, Jonas A, Schulman K (2020). Covid-19 and health Care's digital revolution. N Engl J Med.

[24] Hernández-Quevedo C (2000). Health system response to COVID-19. Eurohealth.

[25] Richardson E, Aissat D, Williams G, Fahy N (2020). Keeping what works: remote consultations during the COVID-19 pandemic. Eurohealth.

[26] F. Funke U-DR. Datenerhebung im Netz: Messmethoden und Skalen. In: M. Welker, O. Wenzel (Hrsg.): Online-Forschung 2007: Grundlagen und Fallstudien. Halem, Köln. 52–76. URL: http://www.frederikfunke.de/papers/2007_onlineforschung.php2007. Cited 11 Nov 2020.

[27] M. T. Thielsch SW. Online-Befragungen in der Praxis. In: T. Brandenburg, M. T. Thielsch (Hrsg.): Praxis der Wirtschaftspsychologie: Themen und Fallbeispiele für Studium und Praxis. Monsenstein und Vannerdat, Münster. 2009. URL: http://www.thielsch.org/download/thielsch_2009_onlinebefragungen.pdf. Cited 11 Nov 2020.

[28] Bund M (2017). Digitales Krankenhaus: große Hoffnungen, ernüchternde Realität.

[29] Mayring P. Qualitative content analysis. Forum Qualitative Sozialforschung / Forum: Qualitative Social Research; Vol 1, No 2 (2000): Qualitative Methods in Various Disciplines I: J Psychol 2000.

[30] Cottrell MA, Russell TG (2020). Telehealth for musculoskeletal physiotherapy. Musculoskelet Sci Pract.

[31] Council of Europe Europaen Union (2020). Social inclusion, digitalization and young people - research study.

[32] Olivero E, Bert F, Thomas R, Scarmozzino A, Raciti IM, Gualano MR (2019). E-tools for hospital management: an overview of smartphone applications for health professionals. Inter J Med Inform.

[33] Sharma S, Oli N, Thapa B (2019). Electronic health-literacy skills among nursing students. Adv Med Educ Pract.

[34] Shaw T, McGregor D, Brunner M, Keep M, Janssen A, Barnet S (2017). What is eHealth (6)? Development of a conceptual model for eHealth: qualitative study with key informants. J Med Internet Res.

[35] Stellefson M, Hanik B, Chaney B, Chaney D, Tennant B, Chavarria EA (2011). eHealth literacy among college students: a systematic review with implications for eHealth education. J Med Internet Res.

[36] Jones JNK, Saunders S (2014). The state of the union: trends and drivers of change in physiotherapy in Ontario in 2014.

[37] Kerst A, Zielasek J, Gaebel W (2020). Smartphone applications for depression: a systematic literature review and a survey of health care professionals’ attitudes towards their use in clinical practice. Eur Arch Psychiatry clinical Neurosci.

[38] Dicianno BE, Parmanto B, Fairman AD, Crytzer TM, Yu DX, Pramana G (2015). Perspectives on the evolution of mobile (mHealth) technologies and application to rehabilitation. J Phys Ther.

[39] Lu K, Marino NE, Russell D, Singareddy A, Zhang D, Hardi A (2018). Use of short message service and smartphone applications in the management of surgical patients: a systematic review. Telemed J E Health.

[40] Jones JNK, Saunders S (2014). The state of the union: trends and drivers of change in physiotherapy in Ontario in 2014.

[41] Filkins BL, Kim JY, Roberts B, Armstrong W, Miller MA, Hultner ML (2016). Privacy and security in the era of digital health: what should translational researchers know and do about it?. Am J Transl Res.

[42] Khan ZF, Alotaibi SR (2020). Applications of artificial intelligence and big data analytics in m-health: a healthcare system perspective. J Healthc Eng.

[43] Escribano B. Digital health: legal challenges in the European Union. Communications law newsletter. International Bar Association, legal practice division. Madrid; 2017. p. 20–23. https://cms.law/es/media/local/cms-asl/files/news-information/press-coverage/ver-articulocompleto5.

[44] Odone A, Buttigieg S, Ricciardi W, Azzopardi-Muscat N, Staines A (2019). Public health digitalization in Europe: EUPHA vision, action and role in digital public health European. J Public Health.

[45] (2008–2013) EHP (2014). Overview of the National Laws on Electronic Health Records in the EU Member States and Their Interaction with the Provision of Cross-border eHealth Services.

