# Supplementary material for: Potential of digitalization within physiotherapy: a comparative survey
Source: BMC Health Serv Res. 2022 Apr 13;22:496. doi: 10.1186/s12913-022-07931-5 (PMC9007581; doi:10.1186/s12913-022-07931-5)
Supplement: Supplementary file 1 — Additional file 1. [file 12913_2022_7931_MOESM1_ESM.pdf]

Evaluation form for physiotherapists on digitalization within physiotherapy

1. General questions (please fill in only for first evaluation)

1. To which age group do you count yourself?

☐ 16-20 ☐ 21-30 ☐ 31-40 ☐ 41-50 ☐ 51-60 ☐ > 60

2. Sex: ☐ female ☐ male

3. Where do you work full-time?

☐ University Hospital
☐ Acute hospital (basic/regular/maximal care)
☐ Rehab hospital
☐ Outpatient rehab center
☐ Employed in practice
☐ Self-employed with practice
☐ Self-employed without practice
☐ Currently still in training
☐ Other (please specify):

2. Digitalization in the professional Environment

4. What is your opinion on the subject of digitization?

☐ I find the topic very interesting and actively prticipate actively in it
☐ The topic is interesting. I take advantage of offers when they fit into my job.
☐ I will wait to use them until I have more experience.are available.
☐ I am rather skeptical about the topic.
☐ I would rather not integrate the topic into my in my job.

5. How "fit" do you consider yourself on the topic of "digitalization" in relation to your professional activities? Please assign yourself a school grade:

☐ Very good ("1") ☐ Good ("2") ☐ Satisfactory ("3") ☐ Sufficient ("4") ☐ Poor ("5") ☐ Insufficient ("6").

6. Do you use smartphone apps in your everyday work (multiple answers possible)?

☐ Yes - for my own organizational support (e.g., patient calendar).
☐ Yes - for my professional support (e.g. AO app, appsfor specific training methods, or similar)
☐ Yes - for communication with colleagues.
☐Yes - for communication with patients (e.g. own practice app, apps for therapy documentation, etc.).
☐ No, I do not want to use apps for my medical work.
Which apps or app functions would you like to have in order to facilitate your work or patient care?
facilitate? _________________

7. What potential do you associate with "digitalization" in the physiotherapy (multiple answers possible)?

☐ Patient care at home/abroad is improved.
☐ The work of physiotherapists is facilitated (e.g., organization, procedures, and processes...).
☐ Communication between individual with collegues is facilitated.
☐ Communication with patients is facilitated.
☐ Measures for disease prevention can be expanded.
☐ Measures for rehabilitation can be expanded.
☐ Others (please specify): _________________

8. What potential problems do you associate with "digitization" in physiotherapy? (Multiple answers possible)

☐ The cost of introducing digital technologies is high
☐ The physical therapist-patient bond is deteriorating.
☐ The practical implementation is not yet mature enough.
☐ I myself have too little experience with the subject.
☐ Data protection is a relevant problem.
☐ The financial remuneration as an incentive is still insufficient
☐ Others (please specify): _________________

9. How do you assess the importance of "big data" or "artificial intelligence" in your professional future?

☐ Big Data and artificial intelligence will significantly shape the way I work.
☐ The topic will increase in relevance.
☐ The topic will not play a relevant role in physiotherapy.

☐ The topic will be irrelevant to my personal work.

10. Which of the following digital services are already used today by you or your employer for your area of work? (Multiple answers possible)

☐ Electronic patient file
☐ Your own homepage
☐ Telemedicine (e.g., video-based communication with Colleagues/Doctors/Patients or similar)
☐ Social media (e.g., Facebook/Instagram/Twitter or similar).
☐ Other (please specify) ________________________
☐ Online video consultation
☐ Electronic appointment scheduling
☐ Email contact option

11. What else occupies/moves you professionally in the context of the topic of "digitization"?

____
____

Thank you very much for your participation!
